# Factors influencing hematological toxicity and adverse effects of perioperative hyperthermic intraperitoneal vs intraperitoneal chemotherapy in gastrointestinal cancer

**DOI:** 10.1515/med-2025-1260

**Published:** 2025-08-29

**Authors:** Xue Zhang, Zhewen Zheng, Hui Gao, Ziqi Yang, Jian Bai

**Affiliations:** Department of General Practice, Beijing Friendship Hospital, Capital Medical University, Beijing, 100050, P. R. China; National Cancer Center/National Clinical Research Center for Cancer/Cancer Hospital & Shenzhen Hospital, Chinese Academy of Medical Sciences and Peking Union Medical College, Shenzhen, 518116, P. R. China; Department of Radiotherapy, Shaanxi Province People’s Hospital, Xi’an, Shaanxi, 710068, P. R. China; Department of General Surgery, Xuanwu Hospital Capital Medical University, Beijing, 100053, P. R. China

**Keywords:** perioperative, hepatotoxicity, intraperitoneal chemotherapy, HIPEC, gastrointestinal cancer, D-dimer

## Abstract

**Background:**

Intraperitoneal (IP) chemotherapy (IPC), including hyperthermic intraperitoneal chemotherapy (HIPEC), has emerged as a promising approach to control peritoneal metastases in gastrointestinal (GI) cancers. However, the safety profile and toxicity spectrum of IPC remain incompletely understood. This study aimed to evaluate the incidence of hematologic and biochemical adverse reactions following surgery with or without IPC and to compare the toxicity profiles of normothermic IPC and HIPEC. Additionally, potential risk factors for liver injury were investigated to guide clinical management.

**Methods:**

In this retrospective cohort study, 449 patients with gastric or colorectal cancer undergoing surgical resection between January 2015 and September 2019 were analyzed. Patients were categorized into three groups: surgery alone (*n* = 171), surgery + normothermic IPC (IPC group, *n* = 82), and surgery + HIPEC (HIPEC group, *n* = 196). Baseline demographic and clinicopathological data, IPC details (including drug regimen, HIPEC technique [open vs closed], and perfusion duration), and postoperative laboratory toxicities were recorded. Hematologic toxicities (leucopenia, neutropenia, thrombocytopenia, and hemoglobin decline) and biochemical toxicities (liver and renal function abnormalities and D-dimer elevation) were graded according to CTCAE v5.0. Group comparisons were performed using *χ* ^2^ or ANOVA tests. Due to a higher proportion of advanced-stage patients in the HIPEC group, stratified analyses were performed by clinical stage (I–II vs III–IV). Logistic regression was used to identify independent risk factors for liver injury in both IPC and HIPEC groups.

**Results:**

Baseline characteristics were comparable across groups except for clinical stage, with the HIPEC group having a higher percentage of advanced-stage patients (79.6 vs 59.8%, *P* <0.05). Compared with the surgery-alone group, both IPC and HIPEC groups had significantly higher incidences of hemoglobin decline (25.7% vs 39.0% vs 49.0%, respectively; *P* <0.01), liver injury (37.4% vs 62.2% vs 60.7%, *P* <0.01), and D-dimer elevation (47.4% vs 68.3% vs 72.9%, *P* <0.01). In contrast, the incidences of leucopenia, neutropenia, and renal impairment were low (<12%) and did not differ significantly among groups. Thrombocytopenia was significantly more frequent in the HIPEC group than in the surgery-alone group (7.7 vs 2.9%, *P* = 0.046). Stratified analyses revealed no significant differences in adverse reaction rates between the IPC and HIPEC groups when adjusted by clinical stage. Multivariate logistic regression indicated that, in the IPC group, severe postoperative GI reactions ( ≥Grade II; OR, 3.72; 95% CI, 1.20–11.55; *P* = 0.023) and the use of a platinum plus docetaxel regimen (OR, 8.75; 95% CI, 1.78–43.12; *P* = 0.008) were independent predictors of liver injury. In the HIPEC group, the platinum plus docetaxel regimen was also associated with higher liver toxicity, and the open HIPEC technique significantly increased the risk (OR 4.80, 95% CI 1.26–18.38, *P* = 0.020).

**Conclusions:**

Both normothermic IPC and HIPEC significantly increase the risk of certain perioperative laboratory abnormalities – specifically, anemia, liver injury, and a hypercoagulable state – compared to surgery alone. Notably, the addition of hyperthermia does not appear to significantly exacerbate the overall toxicity when clinical stage is considered. The chemotherapeutic regimen and HIPEC technique (open vs closed) are key determinants of liver injury. These findings underscore the importance of tailoring IPC protocols and implementing targeted supportive measures, such as liver protection and thromboprophylaxis, to optimize treatment safety in GI cancer patients.

## 1 Introduction

Gastrointestinal (GI) cancers are among the most common malignancies worldwide, ranking third for incidence in men and fourth in women, and their incidence has been rising in recent years [1,2,3]. Early-stage GI cancers are often asymptomatic, and the majority of patients are diagnosed at advanced stages. Advanced tumors frequently involve the serosal layer, leading to a high risk of peritoneal carcinomatosis (PC) and liver metastasis after resection, which contributes to treatment failure and early postoperative recurrence. Even with systemic intravenous chemotherapy, the prognosis of established peritoneal metastasis is extremely poor – the 5-year survival rate of gastric cancer with PC is reported to be essentially 0%, with a median survival under 6 months [1,2].

Cytoreductive surgery (CRS) combined with hyperthermic intraperitoneal chemotherapy (HIPEC) has emerged as an aggressive locoregional treatment for peritoneal metastasis of GI cancers. HIPEC, when added to surgery, can improve the survival and quality of life for selected patients, but it is also associated with a higher incidence of postoperative complications and mortality [3,4]. An alternative approach is intraperitoneal chemotherapy (IPC) administered during or immediately after surgery without hyperthermia (sometimes called normothermic IPC), which relies on the direct cytotoxic effects of drugs on residual tumor cells and is often used in advanced gastric cancer with peritoneal seeding [4]. Both HIPEC and IPC have their advantages, and they remain undetermined which approach is optimal in the perioperative setting for preventing recurrence. Importantly, both treatments have the potential to cause systemic or regional adverse effects, and a direct comparison of their safety profiles and risk factors for toxicity is needed.

Previous studies have highlighted certain complications associated with these treatments. For example, Sleightholm et al. reported that CRS with HIPEC increases the incidence of venous thromboembolism (VTE), suggesting a need for early prophylactic anticoagulation in these patients [5]. Zhu et al. observed that postoperative HIPEC can cause significant hepatic impairment, as well as GI toxicity [6]. Meanwhile, intraoperative IPC (without hyperthermia) has been shown to improve patient survival in colorectal cancer without obvious severe safety concerns [7]. However, to our knowledge, existing studies have neither systematically examined the factors associated with adverse effects of HIPEC and IPC nor directly compared the two modalities in terms of hematological toxicity and related complications.

In this study, we retrospectively analyzed a large cohort of GI cancer patients who received either surgery alone, surgery with IPC, or surgery with HIPEC in the perioperative period. We aimed to compare the incidence of key adverse outcomes (hematologic toxicities, organ function impairments, and hypercoagulability) between these treatment groups and to identify the relevant risk factors contributing to these adverse effects. By elucidating these factors, our goal is to provide guidance on optimizing perioperative IP treatment strategies and managing toxicity to improve patient safety and outcomes.

## 2 Materials and methods

### 2.1 Study participants

This study is a retrospective observational cohort study that included 449 patients with GI malignancies (gastric or colorectal cancer) who underwent surgery at the Department of Oncology, Zhongnan Hospital of Wuhan University between January 2015 and September 2019. Among these patients, 251 had gastric cancer and 198 had colorectal cancer, all confirmed by histopathology. Eligibility criteria for analysis included the following: normal baseline laboratory values for liver function, renal function, coagulation (D-dimer), and blood cell counts before surgery, and no other major abdominal procedures or interventions within 1 month prior to surgery. Patients who had undergone liver surgery, radiofrequency ablation, or neoadjuvant therapy within 1 month before the operation, or those with any pre-existing organ dysfunction, were excluded to ensure a homogeneous baseline for toxicity assessment.

We minimized selection bias through strict eligibility criteria and ensured uniform outcome measurement via standardized laboratory protocols and CTCAE v5.0 grading. Confounders identified in univariate analyses were adjusted in multivariate logistic regression models and stratified analyses by clinical stage controlled for disease severity imbalance. Missing data (<5% for key variables) were excluded in a complete-case analysis, with no significant differences observed between included and excluded cases.

### 2.2 Treatment groups

Patients were divided into three groups based on the perioperative treatment received: surgery + HIPEC, surgery + IPC, and surgery alone as the control. All patients underwent either curative (radical) resection or palliative tumor resection as indicated by their disease.

Surgery + HIPEC: Patients in this group received CRS or tumor resection followed by HIPEC. HIPEC was administered immediately during or after the operation by either an open-abdomen technique (before abdominal closure) or a closed-abdomen technique (after temporary abdominal closure), maintaining a perfusate temperature of 43°C. Agents for HIPEC were selected based on tumor type, peritoneal pharmacokinetics, thermal stability, and documented synergy with hyperthermia. Key considerations included a high peritoneal-to-plasma AUC ratio to maximize local exposure while minimizing systemic toxicity, molecular properties favoring limited systemic absorption, and *in vitro* evidence of enhanced cytotoxicity at 41–43°C. Consequently, platinum-based regimen (oxaliplatin or lobaplatin) combined with docetaxel is preferentially used for gastric cancer, whereas oxaliplatin or lobaplatin with 5-fluorouracil (5-FU) is favored for colorectal cancer, in accordance with published clinical protocols and international guidelines. The duration of hyperthermic perfusion was either ≤60 min or >60 min, depending on the regimen and clinical scenario, with 60–90 min being common for the combined regimens.

Surgery + IPC: Patients in this group underwent surgery followed by IPC without hyperthermia. Chemotherapeutic agents were introduced into the peritoneal cavity intraoperatively or in the early postoperative period at normothermic temperature. The IPC regimens included: 5-FU alone, platinum + docetaxel, or platinum + 5-FU. All Group B patients received their chemotherapy via IP instillation using standard techniques, typically immediately after tumor resection.

**Surgery alone:** Patients in this group received surgical resection (either curative or palliative) with no IPC during the perioperative period.

### 2.3 Baseline characteristics

The main demographic and clinical characteristics of patients in each group were recorded, including age, sex, body mass index (BMI), nutritional risk score, tumor type (gastric vs colorectal), clinical TNM stage, histopathological subtype, smoking status, history of heavy alcohol use, and intraoperative blood transfusion.

### 2.4 Definitions of toxicity outcomes

We defined and categorized postoperative hematological and biochemical toxicities based on clinical diagnostic criteria and international grading standards as follows:

Bone marrow suppression: A decrease in peripheral blood counts postoperatively, specifically leukopenia, anemia, thrombocytopenia, or neutropenia, as measured by routine blood counts. These were noted as present or absent for each patient. We used the Common Terminology Criteria for Adverse Events (CTCAE) v4.0 for grading the severity of cytopenia.

Renal impairment: Postoperative elevation in serum creatinine or blood urea nitrogen beyond the normal range, indicating a decline in renal function. Any degree of acute kidney injury was recorded as an adverse outcome if it met at least CTCAE grade 1 criteria.

Liver function injury: Postoperative elevation in liver enzymes (alanine aminotransferase [ALT] and aspartate aminotransferase [AST]) or bilirubin above the upper limit of normal, consistent with acute drug-induced liver injury. The assessment was based on the diagnostic criteria from the guidelines for diagnosis and treatment of acute drug-induced liver injury, and the severity of hepatotoxicity was graded according to the World Health Organization (WHO) criteria/National Cancer Institute Common Toxicity Criteria (NCI-CTC) version 4.0. For analysis, we primarily considered a binary outcome of any liver function injury vs none and further noted the maximum grade (I–IV) in each patient for descriptive purposes.

D-dimer elevation: An increase in plasma D-dimer level after surgery used as a marker of hypercoagulability and risk for VTE. We categorized D-dimer elevation into two levels: moderate elevation (500–3,500 ng/mL) and high elevation (>3,500 ng/mL), since 500 ng/mL is the typical upper limit of normal. For statistical comparison, any D-dimer >500 ng/mL postoperatively was considered a positive outcome, and we also analyzed factors associated with particularly high elevations (>3,500).

All complications were assessed during the immediate postoperative period and throughout the hospital stay. Laboratory tests were performed for all patients before surgery and monitored after surgery. We ensured that baseline values were normal as part of the inclusion criteria, so any significant abnormality detected postoperatively was attributed to the surgery and/or IP treatment.

### 2.5 Data collection and treatment records

Clinical data were extracted from medical records, including each patient’s treatment details and postoperative course. We recorded whether patients experienced severe GI reactions (nausea/vomiting of grade II or higher according to CTCAE) after the IPC or HIPEC. We also noted intraoperative blood transfusion events and any history of chronic alcohol abuse, as these factors might influence liver function. Nutritional status was evaluated using a standard Nutritional Risk Screening, and BMI was recorded as a continuous variable and categorized (underweight <18.5, normal 18.5–24.9, and overweight ≥25). These factors – GI toxicity, transfusion, alcohol use, nutritional risk, and BMI – were considered as potential contributors to postoperative liver injury or coagulopathy and were included in the analysis of risk factors for complications, particularly in the HIPEC subgroup analysis. We specifically investigated the impact of these variables on the occurrence of liver function injury in the HIPEC group, given prior concerns about hepatotoxicity with HIPEC.

### 2.6 Statistical analysis

All statistical analyses were performed using SPSS version 22.0 (IBM Corp., Armonk, NY, USA). Categorical variables (incidence of specific complications and presence or absence of risk factors) were compared between groups using the chi-square (*χ*²) test or Fisher’s exact test when appropriate. For comparison of the three main groups, pairwise chi-square tests were conducted with a significance level of *P* <0.05. Given the baseline stage imbalance between groups, subgroup analyses stratified by tumor stage (early vs advanced) were also performed for comparison of HIPEC vs IPC outcomes.

To identify independent risk factors for liver injury and for D-dimer elevation, we carried out multivariate logistic regression analyses. Variables that showed significant association with the outcome in univariate chi-square tests were entered into a binary logistic regression model (enter method). For the IPC group and the HIPEC group separately, we examined which factors (chemotherapy regimen, perfusion duration, HIPEC technique, surgical extent, BMI category, presence of severe GI reactions, and tumor pathology) independently predicted liver injury and hypercoagulability. The results of logistic regression are presented as the odds ratio (OR) with 95% confidence interval (CI). A two-tailed *P* value <0.05 was considered statistically significant in all analyses.


**Informed consent:** Given the retrospective nature of the analysis and use of de-identified data, formal written informed consent was waived by the ethics committee. All patients had originally provided consent for their treatment and for the use of their clinical data in research.
**Ethics approval:** This retrospective study was conducted in accordance with the Declaration of Helsinki and was approved by the Ethics Committee of Zhongnan Hospital of Wuhan University.

## 3 Results

### 3.1 Baseline characteristics of patients

The baseline demographic and clinical characteristics of patients were similar across the three groups (surgery alone, surgery + IPC, and surgery + HIPEC). A total of 449 patients were analyzed: 171 in the surgery-only control group, 82 in the surgery + IPC group, and 196 in the surgery + HIPEC group. There were no significant differences between groups in age, sex distribution, tumor type (gastric vs colorectal cancer), pathological type, nutritional risk, smoking status, alcohol use, or incidence of intraoperative blood transfusion (all *P* >0.05). The only baseline variable that differed was clinical stage: the HIPEC group had a higher proportion of advanced-stage (stage III–IV) patients compared to the IPC group (79.6 vs 59.8% advanced stage; *P* <0.05). Due to this imbalance in stage, subgroup analyses stratified by early (stage I–II) vs advanced (stage III–IV) disease were performed for comparison between the IPC and HIPEC groups, as discussed below. Table 1 summarizes the baseline characteristics. All variables except clinical stage show no statistically significant differences across groups.

**Table 1:** Baseline characteristics of the study population by treatment group

Characteristic	Surgery alone (*n* = 171)	Surgery + IPC (*n* = 82)	Surgery + HIPEC (*n* = 196)	*P* (overall)
**Age (years)**	58.6 ± 11.2	59.8 ± 10.5	59.1 ± 11.0	0.72
**Sex, male**	109 (63.7%)	52 (63.4%)	126 (64.3%)	0.98
**BMI (kg/m** ^**2**^)	21.6 ± 2.8	22.6 ± 3.3	21.3 ± 3.7	0.10
**Long-term alcohol use**	37 (21.6%)	15 (18.3%)	40 (20.4%)	0.87
**Smoking history**	46 (26.9%)	20 (24.4%)	55 (28.1%)	0.83
**Nutritional risk (NRS ≥3)**	19 (11.1%)	10 (12.2%)	25 (12.8%)	0.90
**Tumor type**				
Gastric cancer	67 (39.2%)	33 (40.2%)	81 (41.3%)	
Colorectal cancer	104 (60.8%)	49 (59.8%)	115 (58.7%)	
**Pathological type**				
Adenocarcinoma	165 (96.5%)	79 (96.3%)	187 (95.4%)	
Signet-ring/mucinous mix	6 (3.5%)	3 (3.7%)	9 (4.6%)	
**Clinical stage**				
Early (I–II)	49 (28.7%)	33 (40.2%)	40 (20.4%)	
Advanced (III–IV)	122 (71.3%)	49 (59.8%)	156 (79.6%)	
**Intraoperative transfusion**	21 (12.3%)	9 (11.0%)	25 (12.8%)	0.93
**Surgical approach**				
Radical surgery	150 (87.7%)	61 (74.4%)	171 (87.2%)	
Palliative surgery	21 (12.3%)	21 (25.6%)	25 (12.8%)	

Note: Values are presented as count (% of group) or mean ± SD. No significant differences were observed among groups for any variable except clinical stage (advanced vs early-stage distribution differed between the IPC and HIPEC groups, *P* <0.05). IPC = intraperitoneal chemotherapy (normothermic). *P*-values by ANOVA for age (continuous) and the chi-square test for categorical variables.

### 3.2 Incidence of adverse reactions in each group

**Figure 1:**
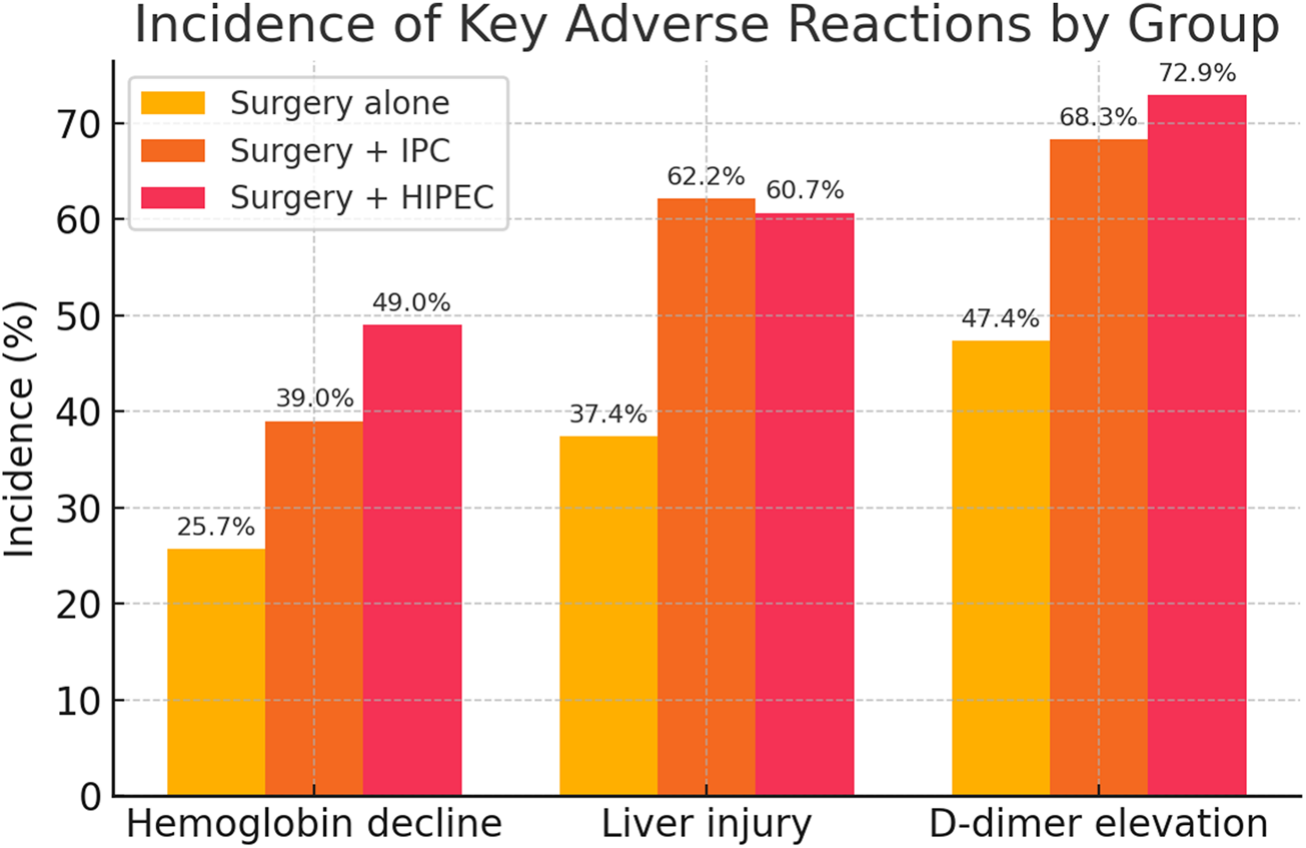
Incidence of key adverse reactions by treatment group.

Major hematologic and biochemical toxicities were compared among the three groups. Table 2 presents the incidence of key adverse reactions (hematologic toxicities and laboratory-defined complications) in each group, along with pairwise chi-square test results comparing the surgery-only group to each treatment group. Overall, patients who received IPC (with or without hyperthermia) experienced higher rates of hemoglobin decline, liver function impairment, and D-dimer elevation compared to surgery alone (*P* <0.01). In contrast, the incidences of leucopenia, neutropenia, and renal impairment were low and did not differ significantly among groups (*P* >0.05). Thrombocytopenia was significantly more frequent in the HIPEC group than in surgery alone (7.7 vs 2.9%, *P* = 0.046), whereas the normothermic IPC group did not differ from controls (2.4 vs 2.9%) (Figure 1). These results indicate that adding IPC, especially with hyperthermia, increases the risk of certain toxicities (notably anemia, liver injury, and hypercoagulability).

**Table 2:** Incidence of key hematologic and biochemical toxicities among the three treatment groups

Adverse reaction	Surgery alone (*n* = 171)	Surgery + IPC (*n* = 82)	Surgery + HIPEC (*n* = 196)
Leucopenia (WBC <4 × 10^9^/L)	2/171 (1.2%)	2/82 (2.4%)	3/196 (1.5%)
Neutropenia (Grade ≥II)	1/171 (0.6%)	3/82 (3.7%)	4/196 (2.0%)
Thrombocytopenia (platelets <100 × 10^9^/L)	5/171 (2.9%)	2/82 (2.4%)	15/196 (7.7%)
Hemoglobin decline (Hb <110 g/L)	44/171 (25.7%)	32/82 (39.0%)	96/196 (49.0%)
Liver injury (elevated ALT/AST or bilirubin)	64/171 (37.4%)	51/82 (62.2%)	119/196 (60.7%)
Renal impairment (Cr or BUN)	19/171 (11.1%)	8/82 (9.8%)	21/196 (10.7%)
D-dimer elevation (>0.5 mg/L)	81/171 (47.4%)	56/82 (68.3%)	143/196 (72.9%)

Note: IPC = intraperitoneal chemotherapy. Postoperative liver injury: any elevation of ALT, AST, or bilirubin above the upper limit of normal, and severity was graded per CTCAE criteria (Grade I–IV). D-dimer elevation: exceeding 0.5 mg/L, which is the standard upper limit of normal in our laboratory.

### 3.3 Comparison of HIPEC and normothermic IPC

Because the HIPEC group had more advanced-stage patients, a stratified analysis was done to directly compare adverse event rates between the two chemotherapy groups within early-stage and advanced-stage subsets. Table 3 shows the incidence of each adverse reaction in the IPC vs HIPEC groups, stratified by the clinical stage (I–II vs III–IV). In early-stage patients, there were no statistically significant differences between IPC and HIPEC in any adverse reaction rate; similarly, among advanced-stage patients, the two treatments showed no significant differences. In early-stage cases, the liver injury incidence was 54.5% (IPC) vs 50.0% (HIPEC), and in advanced cases, 65.3 vs 62.8%, with no significant difference. These results suggest that hyperthermia itself did not significantly increase toxicity compared to normothermic IPC, once clinical stage was accounted for. Consequently, subsequent analyses combined both IPC modalities when compared to surgery alone, and no further direct IPC-vs-HIPEC comparisons were made beyond this point.

**Table 3:** Comparison of adverse reaction incidence between surgery + HIPEC and surgery + IPC groups, stratified by clinical stage

Adverse reaction	Early stage (I–II) IPC vs HIPEC	*P*	Advanced stage (III–IV) IPC vs HIPEC	*P*
Leucopenia	3.0 vs 5.0%	0.6	2.0 vs 1.3%	0.75
Neutropenia	0 vs 2.5%	0.33	4.1 vs 2.0%	0.4
Thrombocytopenia	0 vs 2.5%	0.33	3.1 vs 8.2%	0.2
Hemoglobin decline	42.4 vs 47.5%	0.64	73.5 vs 50.7%	0.08
Liver injury	54.5 vs 50.0%	0.72	65.3 vs 62.8%	0.75
Renal impairment	6.1 vs 7.5%	0.8	12.2 vs 11.3%	0.88
D-dimer elevation	63.6 vs 67.5%	0.75	71.4 vs 74.2%	0.7

Note: Comparison of adverse reaction rates between HIPEC and IPC groups, stratified by clinical stage (early I–II; advanced III–IV).

### 3.4 Severity of liver injury and D-dimer elevation

Both IPC and HIPEC not only increased the incidence of liver injury but also influenced its severity. Table 4 displays the distribution of liver injury severity in each group among patients who experienced liver toxicity. The majority of liver injuries were mild (Grade I) in all groups, but the IPC group had a higher proportion of moderate (Grade II) and severe (Grade III) liver injury compared to the HIPEC group. Specifically, for the patients with liver injury, 29.4% in the IPC group had Grade II–III injury versus 20.2% in the HIPEC group, indicating somewhat more severe liver enzyme elevations with normothermic IPC. However, severe Grade III injury was relatively uncommon overall (2–3% of the patients). The surgery-only group, while having fewer liver injuries overall, showed a similar distribution of mostly mild cases. These data suggest that HIPEC tends to cause predominantly mild liver enzyme elevations, whereas normothermic IPC may more frequently lead to moderate increases.

**Table 4:** Severity of liver injury among patients with liver toxicity in each group

Group	Patients with liver Injury (% of group)	Grade I	Grade II	Grade III	Grade IV
Surgery + HIPEC (*n* = 196)	119 (60.7%)	95 (48.5%)	20 (10.2%)	4 (2.0%)	0
Surgery + IPC (*n* = 82)	51 (62.2%)	36 (43.9%)	13 (15.9%)	2 (2.4%)	0
Surgery alone (*n* = 171)	64 (37.4%)	45 (26.3%)	15 (8.8%)	4 (2.3%)	0

Note: Liver injury graded per CTCAE: Grade I (mild), II (moderate), and III (severe). Severity distribution of liver injury among patients with liver toxicity in each group.

Similarly, we examined the severity of D-dimer elevation. As shown in Table 5, both IPC and HIPEC groups had a high incidence of D-dimer elevation, with a subset of patients reaching markedly high D-dimer levels (>3.5 mg/L) associated with increased risk of thrombosis. The HIPEC group had the highest overall D-dimer elevation rate (72.9% of the patients, vs 68.3% IPC and 47.4% surgery) and also a slightly higher proportion of very high D-dimer (>3.5 mg/L) cases (5.6 vs 4.9% in IPC, and only 1.2% in controls).

**Table 5:** Incidence and severity of D-dimer elevation

Group	Patients with D-dimer ↑ (%)	Mild elevation (0.5–3.5 mg/L)	Marked elevation (>3.5 mg/L)
Surgery + HIPEC (*n* = 196)	143 (72.9%)	132 (67.3%)	11 (5.6%)
Surgery + IPC (*n* = 82)	56 (68.3%)	52 (63.4%)	4 (4.9%)
Surgery alone (*n* = 171)	81 (47.4%)	79 (46.2%)	2 (1.2%)

Note: D-dimer graded as mild (0.5–3.5 mg/L) or marked (>3.5 mg/L). Incidence and severity of D-dimer elevation postoperatively. Mild: 0.5–3.5 mg/L; marked: >3.5 mg/L.

### 3.5 Risk factor analysis for liver injury

We performed further analyses to identify factors associated with the development of liver injury in patients receiving IPC. Separate univariate analyses were conducted for the IPC and HIPEC groups. In the IPC group, the only baseline factor significantly associated with liver injury was the severity of postoperative GI reaction: patients who experienced grade II or worse GI toxicity had a higher incidence of liver injury (76.5 vs 46.2% in those with no or mild GI symptoms; *P* <0.05). Other variables such as age, sex, BMI, tumor type, and surgical approach (radical vs palliative) were not significantly related to liver injury in the IPC group (*P* >0.05). In the HIPEC group, the only baseline characteristic correlating with liver injury was patient BMI: those with BMI <18.5 (underweight) were more likely to develop liver enzyme elevation than those with BMI ≥18.5 (85.7% vs ∼60%; *P* <0.05). This suggests poor nutritional status (low BMI) predisposed patients to hepatotoxicity after HIPEC. No other baseline factors (including the GI reaction, which was not significant in HIPEC) showed significant association with liver injury in the HIPEC group.

In addition to baseline characteristics, we evaluated treatment-related factors. In the IPC group, the IPC regimen was a significant determinant of liver injury (*P* <0.05). Specifically, patients receiving a combination of platinum + docetaxel intraperitoneally had the highest liver injury rate (81.8%), compared to those receiving 5-FU alone (48.9%) or platinum + 5-FU (69.2%). This regimen effect was confirmed in multivariate analysis. In contrast, the type of surgery (radical vs palliative) did not significantly affect liver injury incidence in the IPC group.

**Figure 2:**
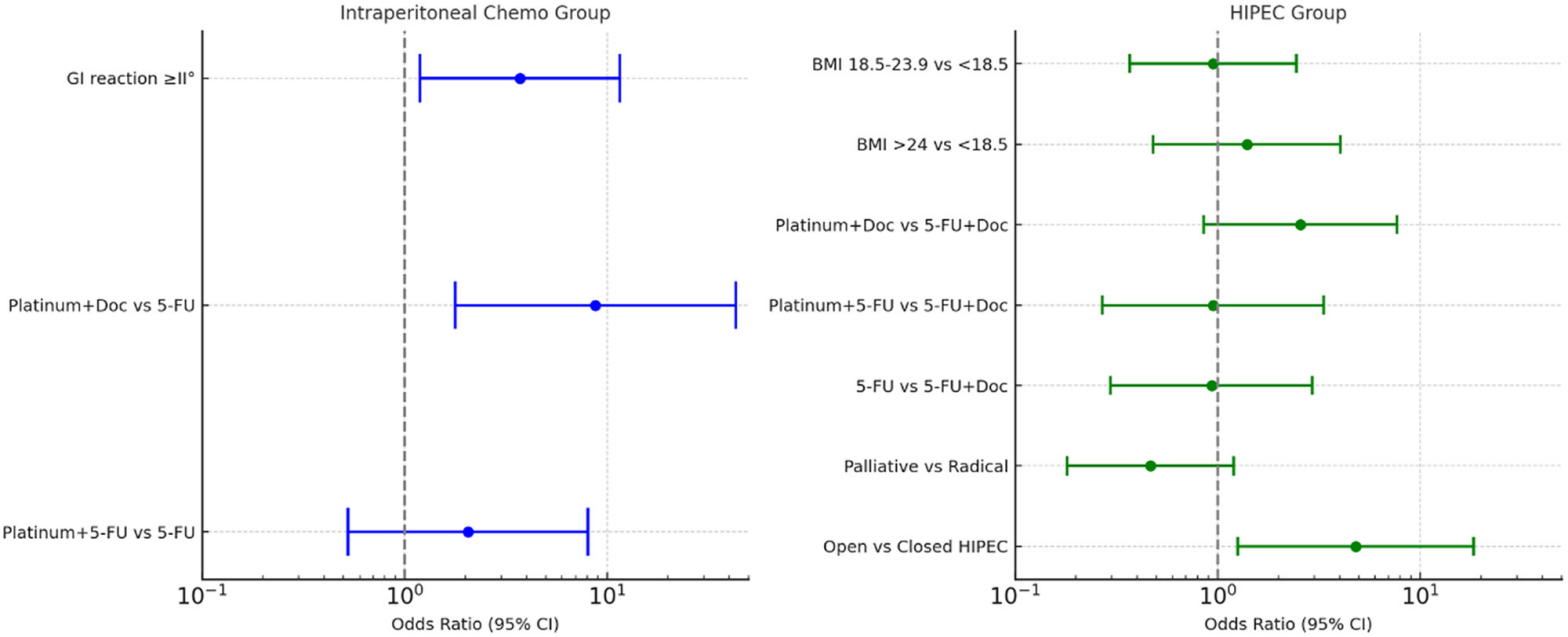
Forest plot of multivariate OR for risk factors of liver injury.

In the HIPEC group, several treatment factors were identified on univariate analysis as related to liver injury: HIPEC regimen, HIPEC technique (open vs closed), duration of perfusion, and surgical extent all showed significant association (*P* <0.05). Platinum + docetaxel again emerged as the HIPEC regimen with the highest liver injury incidence (73.0% vs ∼45–49% for other regimens), open-method HIPEC resulted in more liver injury than closed (74.7 vs 55.2%), and longer perfusion time (>60 or >90 min) was linked to higher liver injury rates than shorter durations. Additionally, patients who underwent more extensive surgery (cytoreductive or major surgery with HIPEC) had a higher incidence of liver toxicity than those who had palliative surgery + HIPEC. These factors were subsequently included in a multivariate logistic regression for the HIPEC cohort. Multivariate logistic regression was performed for each group, incorporating the significant factors from univariate analysis (Tables 6, 7 and Figure 2).

**Table 6:** Multivariate logistic regression for factors associated with liver injury in the IPC group

Factor (IPC group)	OR (95% CI)	*P* value
≥II° GI reaction (vs none/mild)	3.715 (1.195–11.550)	0.023
IP regimen	—	0.024
Platinum + docetaxel vs 5-FU	8.75 (1.776–43.12)	0.008
Platinum + 5-FU vs 5-FU	2.06 (0.527–8.02)	0.299

Note: OR = odds ratio; CI = confidence interval. The reference (OR 1.0) for GI reaction is no or Grade I reaction.

**Table 7:** Multivariate logistic regression for factors associated with liver injury in the HIPEC group

Factor (HIPEC group)	OR (95% CI)	*P* value
BMI <18.5 vs 18.5–23.9 (underweight)	1.00 (reference)	—
BMI 18.5–23.9 vs <18.5 (normal)	0.951 (0.37–2.447)	0.917
BMI >24 vs <18.5 (overweight)	1.396 (0.482–4.045)	0.539
HIPEC regimen overall (4 categories)	—	0.031
Platinum + docetaxel vs 5-FU + docetaxel	2.57 (0.86–7.70)	0.092
5-FU (alone) vs 5-FU + docetaxel	0.94 (0.30–2.95)	0.91
Platinum + 5-FU vs 5-FU + docetaxel	0.95 (0.27–3.35)	0.938
Surgical approach: palliative vs radical	0.467 (0.181–1.200)	0.114
HIPEC method: open vs closed	4.803 (1.255–18.382)	0.02
Perfusion duration >60 min vs ≤60 min	1.375 (0.650–2.908)	0.405

## 4 Discussion

Most patients with GI cancer present at advanced stages, often with tumor invasion beyond the primary organ and a high risk of peritoneal dissemination. Peritoneal metastasis (carcinomatosis) is a common pattern of failure in both gastric and colorectal cancer, occurring in a significant fraction of patients either at diagnosis or as a recurrence after surgery [3,8]. For instance, the incidence of synchronous PC in gastric cancer can be around 14% and for colorectal cancer about 7% [3,8]. Even after an apparently curative resection, microscopic residual disease can seed the peritoneum, which is a sanctuary site due to the peritoneum–plasma barrier that limits systemic chemotherapy penetration [3,8]. To address this, perioperative IPC has been employed as an adjunct to surgery: by delivering chemotherapy directly into the abdominal cavity at the time of minimal residual disease, it aims to eradicate microscopic tumor implants and prevent peritoneal recurrence [9,10,11,12].

This study systematically analyzed the toxicity profile associated with postoperative IPC in patients with GI cancers and investigated the impact of different IPC modalities and regimens on adverse reactions. The main findings include the following: first, compared with surgery alone, the administration of chemotherapy into the peritoneal cavity – regardless of whether it is delivered with hyperthermia – significantly increases the incidence of abnormalities in several laboratory parameters, notably anemia, liver function impairment, and a hypercoagulable state. This suggests that, while IPC improves local tumor control, it also imposes an additional systemic toxic burden. In the HIPEC group, nearly half of the patients experienced a postoperative decline in hemoglobin, compared with approximately one-quarter in the surgery-alone group. The high incidence of anemia may be partially attributed to mild bone marrow suppression induced by IPC or surgical blood loss. Notably, we did not observe a significant increase in leukopenia or neutropenia; the incidences of leukocyte and neutrophil count reductions remained between 1 and 3% across all three groups, with no statistically significant differences. This indicates that the systemic marrow toxicity of IP drug delivery is minimal, which is consistent with previous reports, suggesting that IP administration can reduce the systemic toxicity typically seen with intravenous chemotherapy. In contrast, the impact of IPC on platelets was more evident when hyperthermia was added: the incidence of thrombocytopenia in the HIPEC group was significantly higher than that in the surgery-alone group (7.7 vs 2.9%, *P* < 0.05), while the normothermic IPC group was comparable to the control [13]. This finding suggests that hyperthermia may, to some extent, exacerbate platelet consumption or dilution. However, overall, adverse reactions related to bone marrow suppression remain rare in patients receiving IPC, with most cases being classified as CTCAE Grade 1 and no severe (Grade 3–4) hematologic toxicities observed [14]. Moreover, the incidence of renal impairment was less than 12% in all groups, with no significant differences among them. This observation is in line with recent improvements in HIPEC protocols; previous studies have also reported that modern HIPEC rarely causes severe nephrotoxicity [15]. Overall, our results confirm that the toxic effects of IPC predominantly manifest in hematologic and biochemical parameters – particularly changes in liver function and coagulation – while the bone marrow suppression commonly seen with systemic chemotherapy is not prominent with IP administration.

Second, our study compared the toxicity profiles of HIPEC and IPC. In the unstratified analysis, certain adverse reactions, such as thrombocytopenia and anemia, appeared more frequent in the HIPEC group than in the IPC group. However, since the HIPEC group had a higher proportion of advanced-stage patients (with 79.6% in stage III–IV compared to 59.8% in the IPC group), we performed stratified analyses to control for the effect of tumor stage. The stratified results showed that, among early-stage patients (stage I–II), there was no statistically significant difference in the incidence of adverse reactions between HIPEC and normothermic IPC; similarly, in advanced-stage patients (stage III–IV), the two treatment modalities did not differ significantly. For example, in early-stage cases, the incidence of liver injury was comparable between the HIPEC group and the IPC group (50.0 vs 54.5%), and in advanced-stage cases, the rates were similarly close (62.8 vs 65.3%), with no significant differences observed [16]. These results suggest that, after controlling for disease stage and other factors, hyperthermia itself does not significantly increase IPC-related toxicity. In other words, the safety profiles of HIPEC and normothermic IPC are comparable. This conclusion is consistent with some previous studies; for example, a matched control study in patients with colorectal PC found similar rates of perioperative complications between HIPEC and sequential normothermic IPC, while HIPEC demonstrated better disease control.

We conducted an in-depth analysis of risk factors for liver function impairment, a key toxicity of IPC. Among all patients receiving IPC, liver toxicity was the most common biochemical adverse reaction (with an overall incidence of approximately 61%), and most cases were transient and mild. Through separate logistic regression analyses for the IPC and HIPEC groups, we found that the predictors of liver injury differed between the two. In patients receiving normothermic IPC, the severity of postoperative GI reactions was significantly associated with liver injury: patients who experienced Grade II or higher nausea and vomiting had a liver enzyme elevation incidence of up to 76.5%, which was markedly higher than the 46.2% observed in patients with no or mild GI symptoms. Multivariate analysis confirmed that a GI reaction of ≥Grade II was an independent risk factor for liver injury in the IPC group (OR ≈ 3.72, *P* < 0.05). This phenomenon may reflect a relationship between severe GI toxicity and systemic inflammatory responses or impaired drug metabolism – intense nausea, vomiting, and GI dysmotility can affect nutritional and fluid balance, thereby compromising the liver’s ability to tolerate chemotherapeutic agents. Additionally, in the IPC group, the choice of chemotherapeutic regimen significantly influenced liver toxicity. We observed that patients receiving a combination of platinum and docetaxel had the highest incidence of liver injury (81.8%), whereas those receiving 5-FU monotherapy had the lowest (48.9%), with the platinum plus 5-FU regimen falling in between (69.2%). Multivariate logistic regression further confirmed the importance of regimen differences: compared with 5-FU alone, and the platinum plus docetaxel regimen increased the risk of liver injury by approximately 8.75-fold (OR = 8.75; *P* = 0.008). In contrast, the platinum plus 5-FU regimen did not differ significantly from 5-FU monotherapy, suggesting that regimens containing taxanes (e.g., docetaxel) are more hepatotoxic when administered intraperitoneally [17]. This result aligns with previous literature reporting that the HIPEC regimen is a major determinant of liver toxicity, particularly with cisplatin plus docetaxel significantly increasing postoperative transaminase elevations. Docetaxel, a taxane that undergoes hepatic metabolism, has been associated with elevated liver enzymes in systemic chemotherapy. IP administration of docetaxel results in high local concentrations within the peritoneum and portal circulation, leading to direct hepatic insult.

In the HIPEC group, a broader range of factors – including the chemotherapeutic regimen, HIPEC technique (open vs closed), perfusion duration, and extent of surgery – were associated with liver injury in univariate analysis. Multivariate regression highlighted that the IPC regimen remained an independent predictor of liver injury, with the platinum plus docetaxel regimen conferring the highest risk; in addition, open HIPEC significantly increased the risk compared with closed HIPEC (OR ≈ 4.80, *P* = 0.020). Open HIPEC, typically used in patients with extensive peritoneal dissemination requiring aggressive CRS, likely results in greater exposure of the peritoneal surfaces and adjacent liver tissues to the chemotherapeutic agents, thereby explaining the increased toxicity [17]. Notably, while low BMI (i.e., <18.5) was associated with a higher incidence of liver enzyme elevation in univariate analysis of the HIPEC group, it did not retain significance in the multivariate model, possibly due to interactions with other treatment-related factors [18].

Regarding the potential mechanisms and biological significance of our findings, the observed predominance of liver and coagulation abnormalities following IPC can be attributed to the unique pharmacokinetics of IP drug delivery [17]. When chemotherapy is administered directly into the peritoneal cavity, a substantial proportion of the drug is absorbed via the peritoneal capillaries and enters the portal circulation, thereby exposing the liver to high drug concentrations – a phenomenon known as the “first-pass” effect. This results in transient elevation of liver enzymes, even though the overall systemic absorption is limited. Moreover, the surgical trauma and the inflammatory response induced by both major surgery and hyperthermia can further contribute to transient hepatic dysfunction and activation of the coagulation cascade [19,20]. Our observation of elevated D-dimer levels in the IP chemotherapeutic groups, particularly in the HIPEC group, supports the notion that these patients develop a hypercoagulable state [21]. Although we did not directly correlate these laboratory findings with clinical thromboembolic events, the high incidence of D-dimer elevation emphasizes the need for vigilant postoperative thromboprophylaxis.

The toxicities associated with IPC stem from a combination of local pharmacokinetics, systemic exposure via the peritoneal–portal circulation, and procedure-related stressors. Chemotherapeutic agents administered into the peritoneal cavity achieve high local concentrations, but a fraction is absorbed through the peritoneal capillaries into the portal vein, exposing the liver to a transient high “first-pass” drug load. This leads to elevations in ALT/AST and bilirubin, especially when agents such as platinum compounds and taxanes – which undergo hepatic metabolism – are used. Hyperthermia (41–43°C) increases membrane permeability and enhances drug uptake by peritoneal tumor cells, but it may also exacerbate damage to mesothelial and vascular endothelium, contributing to mild thrombocytopenia and inflammatory cytokine release. Major surgery and HIPEC trigger a surge of pro-inflammatory cytokines, which can impair marrow function and activate coagulation pathways, explaining the observed D-dimer elevations and, to a lesser extent, transient declines in blood cell counts. An open-abdomen HIPEC technique and longer perfusion times increase the peritoneal surface and hepatic exposure, elevating the risk of both liver injury and inflammatory-mediated coagulopathy.

Inter-individual differences in chemotherapy tolerance and toxicity are increasingly attributed to molecular biomarkers. For example, mitochondrial-associated long non-coding RNAs have been implicated in drug resistance mechanisms in laryngeal carcinoma, suggesting that similar lncRNA signatures may underlie variability in hepatotoxicity and hematologic suppression after IPC [22]. Nutritional and metabolic status also play key roles: a recent Mendelian randomization analysis demonstrated that circulating micronutrient levels – including vitamins D and B12 – modulate cancer outcomes, supporting our observation that low BMI predisposes to liver injury post-HIPEC [23]. Moreover, routine biochemical markers such as serum calcium and D-dimer have been shown to predict survival in colorectal cancer, underscoring the value of laboratory indices as early warning signals for adverse events [24]. Preoperative risk stratification using the modified Glasgow Prognostic Score and immune-inflammatory indices has further refined prognostic modeling in GI malignancies [25]. Finally, composite hematologic indices – such as neutrophil-to-lymphocyte ratio combined with platelet-to-lymphocyte ratio – have demonstrated enhanced prognostic accuracy in recent retrospective cohorts [26,27], pointing toward future development of integrated risk models for IPC toxicity.

The clinical implications of our findings are significant. Our results indicate that while IPC – whether delivered under normothermic conditions or as HIPEC – provides a valuable strategy for local tumor control, it also increases the risk of specific toxicities, particularly hepatic injury and coagulation abnormalities. Importantly, the addition of hyperthermia does not seem to substantially exacerbate toxicity when differences in clinical stage are taken into account. Rather, the choice of chemotherapeutic regimen (with platinum plus docetaxel being the most toxic) and technical factors such as the HIPEC method (open vs closed) are the primary determinants of adverse reactions. Clinicians should therefore consider modifying IPC protocols for high-risk patients, such as selecting less hepatotoxic drug combinations and optimizing HIPEC techniques, as well as implementing rigorous perioperative monitoring and supportive measures to mitigate these risks.

Our retrospective cohort design has inherent limitations, including potential selection bias, unmeasured confounding, and inability to infer causality. While strict eligibility criteria, stratification by clinical stage, and multivariate adjustment mitigate these biases, prospective studies are warranted to confirm these findings and explore additional factors such as genetic polymorphisms.

## 5 Conclusions

In conclusion, our study demonstrates that although IPC significantly increases the incidence of laboratory toxicities – particularly anemia, liver enzyme elevations, and D-dimer abnormalities – in GI cancer patients compared to surgery alone, the toxicities are generally mild to moderate and manageable. Moreover, hyperthermic delivery (HIPEC) does not substantially worsen the toxicity profile compared with normothermic IPC when disease stage is controlled. The key determinants of liver injury are the chemotherapeutic regimen, especially the inclusion of taxanes with platinum agents, and the HIPEC technique, with open HIPEC posing a higher risk. These findings provide a rationale for individualizing IPC protocols to maximize therapeutic benefits while minimizing toxicity, thereby improving the overall safety and efficacy of treatment in GI cancer patients.

## References

[1] Cashin PH, Graf W, Nygren P, Mahteme H. Cytoreductive surgery and intraperitoneal chemotherapy for colorectal peritoneal carcinomatosis: Prognosis and treatment of recurrences in a cohort study. Eur J Surg Oncol (EJSO). 2012;38(6):509–15. 10.1016/j.ejso.2012.03.001 22475555

[2] Wei J, Wu ND, Liu BR. Regional but fatal: Intraperitoneal metastasis in gastric cancer. World J Gastroenterol. 2016;22(33):7478–85. 10.3748/wjg.v22.i33.7478 PMC5011663 27672270

[3] Thomassen I, Gestel Y, Ramshorst BV, Luyer M, Bosscha K, Nienhuijs SW, et al. Peritoneal carcinomatosis of gastric origin: A population-based study on incidence, survival and risk factors. Int J Cancer. 2014;134(3):622–8. 10.1002/ijc.28373 23832847

[4] Sun J, Song Y, Wang Z, Gao P, Chen X, Xu Y, et al. Benefits of hyperthermic intraperitoneal chemotherapy for patients with serosal invasion in gastric cancer: a meta-analysis of the randomized controlled trials. BMC Cancer. 2012;12(1):1–10. 10.1186/1471-2407-12-526 PMC3551633 23153379

[5] Sleightholm R, Watley D, Wahlmeier S, Patel A, Foster JM. The efficacy of dextran-40 as a venous thromboembolism prophylaxis strategy in cytoreductive surgery and hyperthermic intraperitoneal chemotherapy. Am Surg. 2017;83(2):134–40. 28228199

[6] Zhu L, Xu Y, Shan Y, Zheng R, Wu Z, Ma S. Intraperitoneal perfusion chemotherapy and whole abdominal hyperthermia using external radiofrequency following radical D2 resection for treatment of advanced gastric cancer. Int J Hyperth. 2019;36(1):403–7. 10.1080/02656736.2019.1579372 30829551

[7] Shang A, Wang S, Yang Y, Li L, Zhao Z, Li D, et al. Effect and safety of intraoperative intraperitoneal chemotherapy on patients suffering from colorectal cancer. World J Surg Oncol. 2021;19(1):84. 10.1186/s12957-021-02197-3 PMC7986417 33752702

[8] Quere P, Facy O, Manfredi S, Jooste V, Faivre J, Lepage C, et al. Epidemiology, management, and survival of peritoneal carcinomatosis from colorectal cancer: A population-based study. Dis Colon Rectum. 2015;58(8):743–52. 10.1097/DCR.0000000000000412 26163953

[9] Lu Z, Wang J, Wientjes MG, Au LS. Intraperitoneal therapy for peritoneal cancer. Future Oncol. 2010;6(10):1625–41. 10.2217/fon.10.100 PMC3076138 21062160

[10] Jian-Yu WU, Hou MX, Cheng HD, Jia LL. Advances in intraperitoneal chemotherapy for gastrointestinal tumors. World Latest Med Inf. 2019.

[11] Zhang J, Guo Y, Wang W, Jing LI, Peng HE, Li-Shu YU, et al. Effect of laparoscopic radical gastrectomy and open radical gastrectomy on inflammatory reaction and immune function in patients with gastric cancer. Chin J Clin Oncol Rehabil. 2016.

[12] Meng LJ, Zhang XR, Yang BJ. Meta analysis on the therapeutic effect of gastric cancer with fluorouracil implants. Chin J Curr Adv Gen Surg. 2016.

[13] Amira G, Morsi A, Fayek IS, Mansour O, Nader H. Hyperthermic intraperitoneal chemotherapy versus systemic chemotherapy in recurrent platinum-sensitive ovarian cancer NCI case control study. Asian Pac J Cancer Prev: APJCP. 2019;20(2):621–7. 10.31557/APJCP.2019.20.2.621 PMC6897027 30806069

[14] Luk KH, Hulse RM, Phillips TL. Hyperthermia in cancer therapy. West J Med. 1980;132(3):179. PMC1272016 7376656

[15] Somashekhar SP, Yethadka R, Kumar CR, Ashwin KR, Zaveri S, Rauthan A. Toxicity profile of chemotherapy agents used in cytoreductive surgery and hyperthermic intraperitoneal chemotherapy for peritoneal surface malignancies. Eur J Surg Oncol: J Eur Soc Surg Oncol Br Assoc Surg Oncol. 2020;46(4 Pt A):577–81. 10.1016/j.ejso.2019.10.032 31677939

[16] Cashin PH, Graf W, Nygren P, Mahteme H. Intraoperative hyperthermic versus postoperative normothermic intraperitoneal chemotherapy for colonic peritoneal carcinomatosis: A case–control study. Ann Oncol. 2012;23(3):647–52. 10.1093/annonc/mdr301 21685413

[17] Zheng Z, Yu H, Xiong B, Shen S, Yang H, Zhou Y. The incidence and risk factors of hepatotoxicity induced by perioperative hyperthermic intraperitoneal chemotherapy in gastrointestinal carcinoma patients: a retrospective study. OncoTargets Ther. 2018;11:5715–22. 10.2147/OTT.S170398 PMC6140724 30254464

[18] Abdel-Rahman O. Effect of body mass index on 5-FU-based chemotherapy toxicity and efficacy among patients with metastatic colorectal cancer; A pooled analysis of 5 randomized trials. Clin Colorectal Cancer. 2019;18(4):e385–93. 10.1016/j.clcc.2019.07.005 31378656

[19] de Witte P, de Witt CA, van de Minkelis JL, Boerma D, Solinger HF, Hack CE, et al. Inflammatory response and optimalisation of perioperative fluid administration during hyperthermic intraoperative intraperitoneal chemotherapy surgery. J Gastrointest Oncol. 2019;10(2):244–53. 10.21037/jgo.2018.12.09 PMC6465482 31032091

[20] Meneses do Rêgo AC, Araújo-Filho I. Assessing the impact of hyperthermic intraperitoneal chemotherapy on anastomotic integrity after cytoreductive surgery in gastrointestinal malignancies. Eur J Clin Med. 2024;5(5):7–13.

[21] Dranichnikov P, Mahteme H, Cashin PH, Graf W. Coagulopathy and venous thromboembolic events following cytoreductive surgery and hyperthermic intraperitoneal chemotherapy. Ann Surg Oncol. 2021;28(12):7772–82. 10.1245/s10434-021-09941-9 PMC8519924 33839978

[22] Wu Z, Chen Y, Jiang D, Pan Y, Tang T, Ma Y, et al. Mitochondrial-related drug resistance lncRNAs as prognostic biomarkers in laryngeal squamous cell carcinoma. Discov Oncol. 2024;15(1):785. 10.1007/s12672-024-01690-x PMC11655928 39692950

[23] Zeng X, Wu Z, Pan Y, Ma Y, Chen Y, Zhao Z. Effects of micronutrients and macronutrients on risk of allergic disease in the European population: A Mendelian randomization study. Food Agric Immunol. 2024;35(1):2442369.

[24] Shu Y, Li KJ, Sulayman S, Zhang ZY, Ababaike S, Wang K, et al. Predictive value of serum calcium ion level in patients with colorectal cancer: A retrospective cohort study. World J Gastrointest Surg. 2025;17(3):102638. 10.4240/wjgs.v17.i3.102638 PMC11948136 40162418

[25] Chen Y, Zhang B, Wang X, Chen Y, Anwar M, Fan J, et al. Prognostic value of preoperative modified Glasgow prognostic score in predicting overall survival in breast cancer patients: A retrospective cohort study. Oncol Lett. 2025;29(4):180. 10.3892/ol.2025.14926 PMC11843409 39990808

[26] Li K, Zeng X, Zhang Z, Wang K, Pan Y, Wu Z, et al. Pan‑immune‑inflammatory values predict survival in patients after radical surgery for non‑metastatic colorectal cancer: A retrospective study. Oncol Lett. 2025;29(4):197. 10.3892/ol.2025.14943 PMC11880885 40046636

[27] Wang K, Li K, Zhang Z, Zeng X, Sulayman S, Ababaike S, et al. Prognostic value of combined NP and LHb index with absolute monocyte count in colorectal cancer patients. Sci Rep. 2025;15(1):8902. 10.1038/s41598-025-94126-7 PMC11909193 40087531

